# What can be learned when multiple analysts arrive at different estimates

**DOI:** 10.1007/s10654-025-01249-2

**Published:** 2025-05-29

**Authors:** Julia M. Rohrer, George Davey Smith, Marcus Munafò

**Affiliations:** 1 Wilhelm Wundt Institute for Psychology, Leipzig University, Leipzig, Germany; 2 Bristol Medical School, University of Bristol, Bristol, UK; 3 MRC Integrative Epidemiology Unit at the University of Bristol, Bristol, UK; 4 School of Psychological Science, University of Bristol, Bristol, UK

Kowall et al. (2025) have brought the multi-analyst approach to epidemiology, with instructive results. With this approach, multiple researchers analyze the same data set to answer the same research question. Or, at least, what *appears* to be the same research question. In fact, the very first paper that popularized this approach [1] has been criticized for using a vague research question (“Are soccer referees more likely to give red cards to players with dark skin tone than light skin tone?”), which apparently led the analysts to try to answer quite different questions—from a simple question about a bivariate statistical association to much more complex causal questions about racial bias [2].

In contrast, the research question passed on to analysts in Kowall et al. (2025) seems less ambiguous: Does marital status influence the incidence of cardiovascular disease? But less ambiguous does not equal unambiguous. Considerable uncertainty surrounding the so-called estimand, the analysis goal, remained, with at least one group explicitly stating that the research question was not focused on causal inference. This highlights the potential ambiguity of words such as “influence” [3], which may only be removed by phrasing the research question in terms of specific counterfactuals or in terms of a hypothetical target trial—but this degree of specificity is rare throughout the literature.

One specific aspect of the estimand is explicitly discussed as a major source of variability by Kowall et al.: They report that while cross-sectional analyses of the longitudinal SHARE data set suggest that those who were never married have a *lower* risk of cardiovascular disease, longitudinal analyses suggest that those who were never married have a *higher* risk of cardiovascular disease. Unfortunately, the authors stop short of precisely articulating how exactly the estimands underlying those estimates vary—are different potential outcomes involved, or are they contrasted in a different manner; are different populations targeted? This only further highlights how the precise articulation of estimands outside of statistical models is new territory. However, the authors do provide one possible explanation for the different signs of the estimates, which points towards different sets of identification assumptions underlying the cross-sectional and the longitudinal analyses. That is, never-married men may be more likely to die after a cardiovascular incident, which means that they may be missed in a cross-sectional snap shot, creating the impression that marriage has adverse effects when in reality it prevents deaths.

Other factors don’t seem to contribute much variability to the estimates. For example, the precise operationalization of marital status or cardiovascular diseases varies between analyses, but that barely seems to matter—as one would hope, given that the marital status groups to be compared and the composite outcome were determined a priori as part of the research question, and given that for both variables, we would expect some ground truth to exist. Reconstructing these pieces of information from the variables available in SHARE may still lead to inconsistencies, but one could expect these to only affect few people relative to the total sample size, and in a rather random manner, minimizing the room for bias.

Much more surprising is the observation that different adjustment sets for confounding also did not make a substantial difference. Adjustment sets varied from the empty set to sets of demographic variables to comprehensive sets including health variables (e.g., self-perceived health). The justifications for the included covariates ranged from none to gut feeling to causal graphs, which is interesting in its own right—it illustrates how messages that statistical adjustment requires causal justification [4] or that some controls are better omitted [5] are not yet universally recognized, even among expert data analysts. But why did it not matter much anyway? After all, not including confounders and including inappropriate confounders should both bias results.

There are different possible explanations here. An optimistic one would be that there just isn’t that much confounding operating between the variables of interest. A pessimistic one would be that there is so *much* confounding operating that even the most extensive adjustment set employed by the analysts is not able to account for it, possibly also due to measurement error in the covariates [6]. One may also argue against the notion that the amount of variability in results is not that big: A relative risk of 1.16 could be considered substantially larger than a relative risk of 1.10. It’s just that in this particular case, this variability is dwarfed by (1) simple sampling error and (2) the substantial discrepancy between cross-sectional and longitudinal analyses.

Speaking of quantification, another issue that remains open is the choice of appropriate metrics when pooling disparate analyses. Ideally, all effect estimates would be expressed in a common metric to ensure they are actually comparable [7]. Kowall and colleagues instead present a range of Hazard Ratios, Odds Ratios and Relative Risks. There are two pragmatic arguments to justify the usage of disparate metrics. First, it may not make a substantial numerical difference in this particular case—if the prevalence is low, Odds Ratios approach Relative Risks according to the rare disease assumption [8]. Second, readers of studies may interpret these different metrics in the same way anyway, thus rendering the differences between them irrelevant in practice.

But they are not irrelevant when the aim is to precisely identify sources of discrepancy, in which case a common effect metric would be desirable. There remains work to be done to enable translation between metrics for very different classes of models—which admittedly may not *always* be possible, but is at least conceivable, in particular when the underlying data are available (rather than just summary statistics). A promising development on that front is work on a more comprehensive *marginaleffects* framework in the social sciences [9] that enables researchers to derive a wide number of effect size quantifications from disparate classes of statistical models (with accompanying software packages in R and Python). This, in turn, should enable us to find out whether two different statistical models give the same answer when asked *precisely* the same question.

It is worth noting that multi-analysts studies have attracted more general criticism, including whether they are worth the effort in the first place. After all, the time and energy of the numerous analysts could have been used on other (original) research [7]. Of course, whether that original research would have been a better investment will surely depend on one’s assessment of the average quality of original research in the field. And, even outside of a multi-analyst project, researchers may end up analyzing overlapping data to answer the same research question, resulting in rather redundant efforts (as an example, witness five different studies all investigating the effects of gut microbiota on erectile dysfunction with the help of Mendelian randomization: [10–14]. More importantly, multi-analysts studies can potentially have positive downstream effects on the quality of original research in the field. But it’s always good to consider opportunity costs, in particular when the labour of so many specialists is involved.

Another argument has been that the results of multi-analysts studies can be uninformative, given that analysts often lack the relevant substantive domain expertise [quoted in e.g. 15]; only results generated by analysts with the relevant expertise should be taken seriously in the first place. But participating analysts quite often do have relevant expertise (as was the case in the present project), and, in any case, the primary scientific literature does not come with any guarantees that data analysts have the relevant expertise—for example, anybody can call themself an epidemiologist (as evident from the proliferation of “epidemiological” studies during the COVI-19 pandemic).

More importantly, this study—as well as other multi-analyst studies [1, 16]—have highlighted that it is a very specific type of expertise that seems to be widely missing: the ability to clearly articulate estimands in precise terms and to find an appropriate model that identifies the estimand under defensible assumptions [17]. What is missing is not necessarily substantive knowledge, but rather the knowledge of how to translate that substantive knowledge into appropriate statistical analyses to answer substantively meaningful questions. And this problem is by no means limited to multi-analyst studies; multi-analyst studies merely highlight a problem that may otherwise remain hidden.

Of course, this issue is well-known among statisticians who advise applied researchers [18]. But, as the last decade of discussions surrounding replicability and reproducibility have highlighted, many problems that have been identified by individuals long ago only get tackled properly once a certain degree of awareness exists.

The study by Kowall and colleagues (2025) will hopefully raise such awareness with their valuable snapshot of current practices in epidemiology, highlighting sources of disagreement and room for future improvement. In the end, the value of multi-analyst studies is not that they provide answers to specified research questions, but that they provide insights into current practices, including their limitations.

If these insights are put to good use by the wider community in the field, they may be well worth the effort exerted by the multiple analysts who contributed.
